# Comparative Analyses of the Safety Profiles of Vitamin D Receptor Agonists: A Pharmacovigilance Study Based on the EudraVigilance Database

**DOI:** 10.3390/ph17121686

**Published:** 2024-12-13

**Authors:** Zsolt Gáll, Melinda Kolcsar

**Affiliations:** Department of Pharmacology and Clinical Pharmacy, Faculty of Pharmacy, George Emil Palade University of Medicine, Pharmacy, Science, and Technology of Targu Mures, 540142 Targu Mures, Romania; melinda.kolcsar@umfst.ro

**Keywords:** vitamin D, vitamin D receptor agonists, safety profile, pharmacovigilance, calcitriol

## Abstract

**Background/Objectives**: Vitamin D receptor (VDR) agonists are commonly used in clinical practice for their roles in calcium regulation and potential benefits in various diseases. However, their safety profiles, particularly for compounds available as food supplements, remain underexplored in real-world settings. This study aimed to analyze the safety profiles of VDR agonists using the EudraVigilance database, focusing on adverse drug reactions (ADRs) reported between 1 January 2004 and 23 June 2024. **Methods**: Data for ten VDR agonists were collected, de-duplicated, and analyzed to identify specific safety signals. Risk factors for specific ADRs were assessed using multiple logistic regression. **Results**: This study analyzed 5,369,581 reports in the EudraVigilance system, from which 17,947 reports (0.33%) involving 80,050 ADRs were linked to VDR agonists. The most-reported drugs were cholecalciferol (12,944 cases) and calcitriol (1355 cases). Serious ADRs were more prevalent with paricalcitol, alfacalcidol, and calcitriol than with cholecalciferol (*p* < 0.05). Hypercalcemia was a hallmark ADR for all VDR agonists, with the highest risk linked to dihydrotachysterol (ROR = 5668; 95%CI = 3332 to 9641; *p* < 0.0001), alfacalcidol (ROR = 965.7; 95%CI = 843.6 to 1106; *p* < 0.0001), and calcitriol (ROR = 726.0; 95%CI = 634.6 to 830.5; *p* < 0.0001). Logistic regression highlighted dehydration, overdose, and concomitant administration of calcium salts as major predictors of hypercalcemia. The co-administration of multiple VDR agonists was also found to increase hypercalcemia risk. However, the disproportionality analysis showed that only active VDR agonists (e.g., calcitriol, alfacalcidol) were associated with severe complications like renal and urinary disorders and cardiac issues due to hypercalcemia. Natural precursors (cholecalciferol, ergocalciferol) were more often linked to non-calcemic ADRs such as gastrointestinal symptoms, which were more prevalent in infants and children compared to adults. **Conclusions**: The safety profiles of VDR agonists differ significantly between compounds. Active derivatives require close monitoring for serious calcemia-related complications, whereas cholecalciferol is associated with less severe ADRs, primarily in at-risk populations. These findings highlight the need for targeted safety monitoring and further research into the real-world uses of VDR agonists.

## 1. Introduction

Vitamin D, traditionally recognized for its pivotal role in calcium homeostasis and bone health, has recently been implicated in a wide array of physiological processes, including immune modulation, cardiovascular health, and cancer prevention [1,2,3,4,5,6,7]. Vitamin D receptor (VDR) agonists/activators were developed and approved as medicines (1) to address deficiencies in the vitamin D synthesis pathway including hydroxylation at 1a and 25 positions, (2) to obtain increased biological activity, or (3) to decrease the hypercalcemic effect compared to calcitriol [8]. In this manner, not only the desired effects could be achieved, but the safety profile might also be enhanced. Although significant effort has been made to develop vitamin D receptor agonists with or without a secosteroidal structure [9,10], only the following vitamin D agonists have been approved by the European Medicines Agency (EMA) or any national agency in the European Union: calcitriol, calcifediol, calcipotriol, cholecalciferol, alfacalcidol, dihydrotachysterol, doxercalciferol, ergocalciferol, paricalcitol, and tacalcitol. Vitamin D and VDR agonists have well-documented benefits, but concerns about their safety profile and potential adverse effects are growing.

In general, VDR agonists are used in conjunction with certain pathological conditions (e.g., osteoporosis, chronic kidney disease, hypoparathyroidism, and secondary hyperparathyroidism) that necessitate their use [1,11,12]. On the other hand, the global burden of vitamin D deficiency highlights the need for proper and safe interventions to mitigate complications. To prevent and treat vitamin D deficiency, cholecalciferol or calcifediol is typically used, depending on the medical condition. The dosing of cholecalciferol (or calcifediol) should be based on serum 25-hydroxy vitamin D concentration [13]. Although the latest guidelines on vitamin D deficiency prevention do not recommend routine serum 25-hydroxy vitamin D testing in the general population—only for at-risk groups—cholecalciferol has become a commonly used dietary supplement in recent years [14]. Due to its increased consumption, it is possible that some VDR agonists are used concomitantly with cholecalciferol, underscoring the need to understand the toxicity thresholds and risks associated with excessive vitamin D intake both as supplement and as a drug.

Vitamin D toxicity due to VDR activation is usually diagnosed based on elevated serum 25-hydroxy vitamin D levels accompanied by hypercalcemia and hypercalciuria [15]. These are caused by an increase in calcium absorption from the intestine as well as increased bone mobilization. However, patients’ complaints might include neuropsychiatric symptoms such as lethargy, confusion, irritability, depression, hallucinations; gastrointestinal symptoms such as decreased appetite, nausea, vomiting, and constipation; cardiovascular manifestations such as bradycardia and arrythmias; and renal symptoms such as polyuria and renal colic associated with nephrolithiasis [16].

Over the past few decades, numerous studies have attempted to define the safe upper limits of vitamin D consumption. Notably, research by Vieth et al. has emphasized the complexities involved in determining these thresholds, suggesting that toxicity is rare but possible at very high doses [17]. Furthermore, studies by Hathcock et al. and Rizzoli have provided comprehensive reviews of the safe upper intake levels, stressing the importance of individualized assessments based on varying physiological needs and health conditions [18,19]. While the risk of developing hypercalcemia and its complications from cholecalciferol supplementation is undeniable, a median dose of 4000 IU of cholecalciferol is considered safe even for long-term use, and has been demonstrated to provide multiple health benefits [13].

Pharmacovigilance, the practice of monitoring the adverse effects of drugs after they have been licensed for use, offers a real-world perspective on the safety of drugs [20,21]. By analyzing pharmacovigilance data, researchers can identify patterns of adverse reactions and potential risk factors that may not be evident in controlled clinical trials [22]. The recent literature increasingly focuses on the safety profile of vitamin D, particularly through the lens of pharmacovigilance databases. For instance, data from adverse event reporting systems have revealed cases of hypercalcemia, renal impairment, and cardiovascular issues associated with high doses of vitamin D [23]. The findings suggest that while vitamin D is generally considered safe, vigilance in monitoring its use is necessary, particularly as supplementation practices evolve.

Furthermore, a systematic review and meta-analysis conducted by Zittermann et al. in 2023 examined the long-term safety of high-dose vitamin D supplementation, specifically doses ranging from 3200 to 4000 IU daily. They highlighted the urgent need for rigorous reporting of safety-related outcomes in vitamin D supplementation trials, particularly given the rising intake levels and recommendations for higher dosages [24]. The analysis indicated that, while adverse events were reported, the overall safety profile remained acceptable, reinforcing the need for continued pharmacovigilance in this area.

The purpose of this study is to leverage real-world pharmacovigilance data to conduct a comprehensive risk assessment of vitamin D agonists. This approach aims to bridge the gap between controlled experimental data and the complex realities of clinical practice, thereby providing a more nuanced understanding of the safety profiles of these compounds. By identifying and analyzing adverse reaction patterns, this study seeks to inform clinicians and optimize the therapeutic use of vitamin D agonists.

## 2. Results

### 2.1. Distribution for Sex, Age, the Type of Reporter, and Country of ICSRs Involving VDR Agonists

All individual case safety reports (ICSRs) were exported from the EudraVigilance database using the line listings option for the whole study period (from 1 January 2004 to 23 June 2024). In total, the 5,369,581 reports included 16,894,422 individual suspected adverse drug reactions (ADRs) reported as preferred terms (PTs). From this dataset, the total number of reports referring to VDR agonists was 17,947, which contained 80,050 ADRs. Not surprisingly, the highest number of reports was found for cholecalciferol and calcitriol, with 12,944 and 1355 ICSRs, respectively. In this order, the two natural compounds were followed by alfacalcidol (1166 ICSRs), ergocalciferol (826 ICSRs), paricalcitol (651 ICSRs), and calcifediol (564 ICSRs). There were 317 cases of calcipotriol, 68 cases of dihydrotachysterol, 34 reported cases of tacalcitol, and only 22 reported cases of doxercalciferol.

Of all the reports, 11,771 (65.59%) were made by healthcare professionals. More than half of the reports came from the European Economic Area (EEA) than non-EEA countries (10,660; 59.40% vs. 7287; 40.60%). As for the sex of patients, 4672 (26.03%) of the reports included information about female patients, whereas 12,609 (70.26%) were from male patients; the remaining reports had no sex specified (666; 3.71%). For detailed information on regional and sex distributions for each VDR agonist, see Supplementary Materials, Tables S1–S3. The majority of the reports included the age group of 18–64 years (5827; 32.47%), followed by those in the group of 65–85 years (5335; 29.73%), while adolescents aged between 12 and 17 years had the lowest percentage (Table 1).

### 2.2. Distribution for Seriousness of ICSRs Involving VDR Agonists

Analyzing the seriousness of the reported ADRs, it was found that 6578 (36.7%) reports had not specified/unknown seriousness. In descending order of seriousness, 1066 (5.94%) cases were reported as resulting in death, 732 (4.08%) as being life-threatening, 297 (1.65%) as disabling, and 3772 (21.02%) as causing/prolonging hospitalization. A very small proportion of 42 (0.23%) cases were categorized as congenital anomalies. The remaining 5451 (30.37%) cases were other medically important conditions. For detailed information on each VDR agonist, see Table 2.

As a percentage of the total number of serious cases (i.e., those that resulted in death, were life-threatening, disabling, and caused/prolonged hospitalizations), paricalcitol (66.67%), alfacalcidol (57.29%), and calcitriol (49.37%) were associated with higher risk than cholecalciferol (25.22%). Surprisingly, ergocalciferol was also associated with a high number of serious cases: 432 of 826 (52.3%) of which 92 (11.14%) were resulting in death.

**Figure 1:**
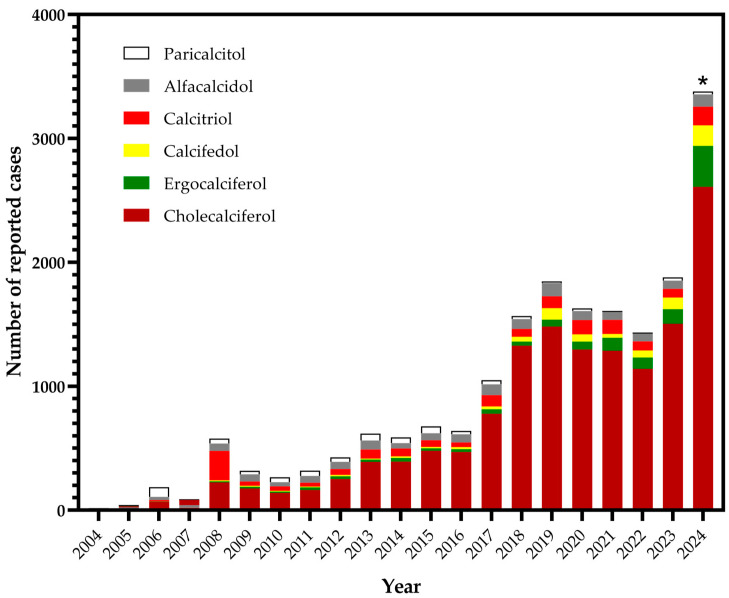
Patterns in adverse drug reaction reporting for vitamin D receptor agonists in the EudraVigilance database over the study period. Asterisk marks that data for this period were obtained by extrapolating the number of cases registered in the first half of the year.

Based on the distribution of cases by year, it was observed that the EudraVigilance database had shown a significant increase in the number of reported cases in the last 20 years, primarily due to the EMA’s efforts. The COVID-19 pandemic had a significant impact on the steady increase until 2020 due to a large number of adverse reactions reported for vaccines and antivirals. Nevertheless, analysis of ICSRs for VDR agonists indicated that cholecalciferol is increasingly being used as a drug, while other VDR agonists have exhibited a constant reporting rate (Figure 1).

### 2.3. Analysis of Reported ADRs and Concomitantly Administered Drugs

#### 2.3.1. Cholecalciferol

The most frequently reported ADR for cholecalciferol was abdominal pain and discomfort, summing up to 2162 of 58,323 (3.71%) ADRs. Further gastrointestinal complaints such as nausea (995; 1.71%), diarrhea (927; 1.59%), vomiting (662; 1.14%), and constipation (557; 0.96%) were also frequently reported. General health issues like fatigue (746; 1.28%), headache (631; 1.08%), choking (609; 1.04%), pain (559; 0.96%), and dyspnea (514; 0.88%) were also prevalent. Dermatological complications were the next important concern, including pruritus (661; 1.13%), rash (543; 0.93%), and urticaria (345; 0.59%). Hypercalcemia was reported in 722 ICSRs (1.24% of all ADRs), but its complications such as nephrolithiasis were reported in only 144 cases (0.25%). Cardiac complications including arrhythmia, bradycardia, tachycardia, and hypertension were observed in 608 (1.04%) cases, while neuropsychiatric conditions such as mental status changes, depression, altered state of consciousness, and anxiety were described in 531 (0.91%) patients (Table 3).

Overall, based on the indications mentioned in ICSRs, the highest number of patients (1640 out of 12,944) used cholecalciferol for the treatment or prevention of osteoporosis. The second most frequent indication was vitamin D deficiency (1370 ICSRs), followed by prophylactic supplementation (1302 cases). A significant proportion of the cases were related to rheumatoid arthritis (705 cases), where the average number of ADRs was 19.14 per patient, four times higher than the total average of 4.51 per patient. In line with the abovementioned indications, the drugs most frequently associated with cholecalciferol were calcium salts (carbonate, glubionate, gluconate), ascorbic acid, pantoprazole, alendronate, methotrexate, adalimumab, leflunomide, etanercept, magnesium salts, and hydrocortisone.

#### 2.3.2. Calcitriol

The most frequently reported ADR for calcitriol was hypercalcemia, reported in 304 of 5755 (5.28%) ADRs. The list of commonly reported ADRs included: nausea, abdominal pain, constipation, off-label use, vomiting, headache, pain, acute kidney injury, and dyspnea (Table 3). Nephrolithiasis was reported in only 26 (0.45%) patients.

The available information on indications and concomitantly used drugs was also analyzed. The frequently mentioned indications included osteoporosis, hypoparathyroidism, hypocalcemia, vitamin D deficiency, and supplementation. Accordingly, the ICSRs mentioned calcium carbonate, magnesium, and potassium salts as the most frequently associated drugs with calcitriol. Of note, the study identified a subgroup of kidney or liver transplant patients (*n* = 22) as the most vulnerable group for ADRs, with 7.23 ADRs reported per patient on average. Based on all 1355 ICSRs combined, the average number of ADRs per patient was 4.24.

#### 2.3.3. Alfacalcidol

The most frequently reported ADR for alfacalcidol was hypercalcemia, being reported for 322 of 3866 (8.33%) ADRs. The list of commonly reported ADRs included nausea, abdominal pain, diarrhea, vomiting, headache, pain, and dyspnea. Renal complications such as acute kidney injury, renal impairment, renal failure, nephrocalcinosis and nephrolithiasis were also frequent (Table 3). Similar to cholecalciferol, alfacalcidol also induced dermatological ADRs such as rash, pruritus, erythema, and urticaria. The most frequently found indications for alfacalcidol were osteoporosis (398 of 1166 cases; 34.1%), rheumatoid arthritis (312; 26.8%), hypocalcemia (155; 13.3%), and hypoparathyroidism (137; 11.7%). Consequently, the most frequently used concomitant drugs were calcium salts (913; 78.3%), alendronate (159; 13.6%), prednisolone (115; 9.86%), and methotrexate (110; 9.43%). Surprisingly, a significant proportion of patients (155 of 1166 reports; 13.3%) were given cholecalciferol along with alfacalcidol, leading to 42 cases of serious hypercalcemia.

#### 2.3.4. Ergocalciferol

The most frequently reported ADR for ergocalciferol was gastrointestinal reactions, with abdominal pain reported in 88 of 7644 (1.2%) ADRs. Nausea, vomiting, diarrhea, and constipation were also reported. Similarly, general reactions such as fatigue, headache, malaise, pain, and dyspnea were also frequent. Renal complications were much less frequent than for other VDR agonists (Table 3). Hypercalcemia (49; 0.64%) was reported in a smaller proportion than for other VDR agonists. However, drug ineffectiveness (116; 1.52%) and the incompleteness of the therapeutic product effect (69; 0.90%) were more prevalent compared to other compounds. The most frequently found indications for ergocalciferol were vitamin supplementation (167 of 826; 20.2%) and prophylaxis (141; 17.1%).

#### 2.3.5. Paricalcitol

The use of paricalcitol was associated with the most severe ADRs, as 181 of 651 (27.8%) reported cases resulted in death. It should be noted that patients benefiting from paricalcitol treatment are polymorbid and polymedicated, so the causality between paricalcitol and the cause of death could not be determined. The most frequently reported ADR was hypercalcemia (40 out of 1865 reported PTs; 2.14%); however, life-threatening conditions potentially linked to paricalcitol treatment were also prevalent: infection (38; 5.84%), dyspnea (36; 5.53%), myocardial infarction (30; 4.61%), sepsis (27; 4.18%), and cardiac failure (20; 3.07%) (Table 3). Other frequently reported PTs were nausea (29; 4.45%), blood parathyroid hormone increased (28; 4.3%), diarrhea (24; 3.69%), and fall (22; 3.38%). The indications were secondary hyperparathyroidism, chronic kidney disease, and renal failure, or it were not reported.

### 2.4. The Most Specific ADRs Reported for VDR Agonists

#### 2.4.1. Hypercalcemia

**Figure 2:**
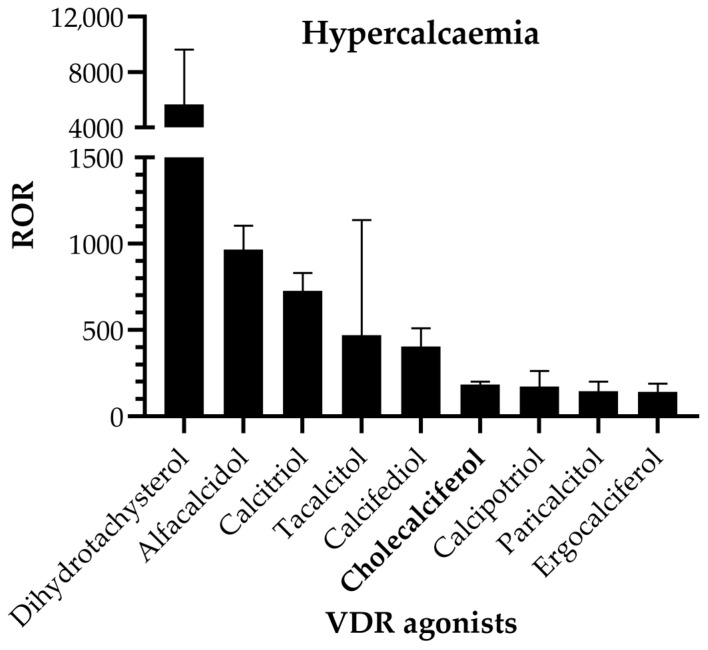
Reporting odds ratio (ROR) for hypercalcemia induced by vitamin D receptor agonists. Data is presented as with ROR with 95% confidence interval for all vitamin D agonists that had the minimum of three cases registered.

Hypercalcemia was reported in 2,442 ICSRs registered in the study period, including all drugs. The association between hypercalcemia and VDR agonists was analyzed by calculating the proportional reporting ratio (PRR) and the reporting odds ratio (ROR) along with 95% confidence interval (95%CI) for each compound that had the minimum of three cases registered. The results showed that the highest risk of developing hypercalcemia was associated with dihydrotachysterol (ROR = 5668; 95%CI = 3332 to 9641; *p* < 0.0001), alfacalcidol (ROR = 965.7; 95%CI = 843.6 to 1106; *p* < 0.0001; PRR = 699.31), and calcitriol (ROR = 726.0; 95%CI = 634.6 to 830.5; *p* < 0.0001; PRR = 563.32) compared to the full reference database (Table 2). Hypercalcemia is a highly specific adverse reaction of all VDR agonists, but the relative risk of hydroxylated precursors was found to be higher than that of cholecalciferol (Figure 2).

After conducting a subgroup analysis by age group and sex, it was found that more than a third of all hypercalcemia cases were reported for females (1446/2442; 59.2%) in the 65–85 years age group (893/2442; 36.6%). A more in-depth analysis of hypercalcemic cases focused on the possible risk factors. Each case was checked for the presence of the following conditions: overdose; concomitant administration of calcium salts; drug interactions with lithium, thiazide, and thiazide-like diuretics; certain comorbidities that affect calcium homeostasis (e.g., cancer, familial hypocalciuric hypercalcemia, dehydration, etc.).

Taking into account all ICSRs registered for cholecalciferol, calcitriol, alfacalcidol, paricalcitol, and ergocalciferol, a logistic regression analysis was performed. The first model was constructed using all hypercalcemic conditions listed above. In this model, dehydration and overdose had the largest effect size, but the administration of calcium salts and lithium were also significant. On the other hand, the presence of different cancer types was a significant negative predictor. Although this model was highly significant (LLR *p*-value: 4.801 × 10^−54^), it explained around 2.86% of the variance in the outcome only (pseudo R-squared 0.02861).

Several models were constructed by introducing the age (divided into vulnerable and non-vulnerable classes), sex, the number of concomitant drugs (more or less than five), and the interactions between the variables (Supplementary Materials, Table S4). The Akaike Information Criterion (AIC) values were used to choose the best-fitting model. Model 4, which included 12 covariates, provided the lowest AIC value and a pseudo R-squared of 0.03615 (LLR *p*-value: 4.303 × 10^−65^). This model showed that the vulnerable age group (<18 and >65 years) was not a significant predictor, while the female sex did show a meaningful negative effect on hypercalcemia (Table 4).

The results presented in Table 4 indicated that dehydration and overdose had the strongest positive influence on hypercalcemia, whereas the concomitant administration of calcium salt, thiazides, and lithium were consistently meaningful predictors, while cancer as comorbidity and polypharmacy (five or more concomitantly administered drugs) showed negative effect. In other words, the odds ratio values calculated by exp (coefficient) showed that those patients which were diagnosed with dehydration and used and overdose of VDR agonist had an odd ratio (95% CI) of 5.05 (2.45–10.4) and 3.62 (2.84–4.62), respectively, to develop hypercalcemia. In contrast, the interaction between age and polypharmacy revealed that patients from the vulnerable age groups are more likely to develop hypercalcemia due to cumulative risks (OR = 2.30; 95%CI = 1.76–3.02). Furthermore, the combination of two or more vitamin D agonists also increased the risk of developing hypercalcemia (OR = 3.89; 95%CI = 3.19–4.73).

#### 2.4.2. Possible Complications of Hypercalcemia

Long-term hypercalcemia may result in severe complications. The widespread use of cholecalciferol for food supplementation and the lack of routine monitoring for calcemia make it important to address the potential complications associated with hypercalcemia. The complications of hypercalcemia include gastrointestinal (e.g., constipation, pancreatitis, gastric ulcers), cardiovascular (e.g., arrhythmias, syncope), neurological (e.g., paresthesias), psychiatric (e.g., altered mental status, depression), and renal disorders (e.g., nephrolithiasis, renal failure). All reported PTs were transformed to SOCs. For each VDR agonist–SOC pair, PRR values were calculated taking into account all ADRs and separately for the serious ADRs (Table 5).

The results confirmed that all VDR agonists were associated with metabolism and nutritional disorders, but cholecalciferol remains the least dangerous. Similarly, the potential renal and urinary complications of hypercalcemia showed higher PRR values for 1α hydroxylated compounds such as calcitriol and alfacalcidol than for cholecalciferol and ergocalciferol. Conversely, gastrointestinal complaints showed higher PRR values for cholecalciferol and ergocalciferol. However, the high PRR values for several SOCs indicated that ergocalciferol may not be as safe as cholecalciferol.

#### 2.4.3. Potential Signals of Serious Risks

Long-term hypercalcemia may result in severe complications. The widespread use of cholecalciferol for food supplementation and the lack of routine monitoring for calcemia make it important to address the potential complications associated with hypercalcemia. The complications of hypercalcemia include gastrointestinal (e.g., constipation, pancreatitis, gastric ulcers), cardiovascular (e.g., arrhythmias, syncope), neurological (e.g., paresthesias), psychiatric (e.g., altered mental status, depression), and renal disorders (e.g., nephrolithiasis, renal failure). All reported PTs were transformed to corresponding system organ class (SOC). For each VDR agonist–SOC pair, PRR values were calculated taking into account all ADRs and separately for the serious ADRs (Table 5).

The PRR values calculated for each VDR agonist–SOC pair revealed some additional associations between ADR classes and compounds. The highest PRR value was observed for Product issues, which might be attributed to the wide usage of cholecalciferol and ergocalciferol as a food supplement. For cholecalciferol, the most frequently reported PTs related to Product issues were “off-label use”, “product use in unapproved indication”, “drug ineffective”, “product use complaint”, and “wrong technique in product usage process”. In the case of ergocalciferol, the highest number of product related PTs included “therapeutic product effect incomplete”, “drug ineffective”, “off-label use”, and “product use in unapproved indication”.

The second most increased PRR value was obtained for pregnancy, puerperium, and perinatal conditions. By analyzing the most frequently reported ADRs it was observed that “exposure during pregnancy”, “maternal exposure during pregnancy”, and “fetal exposure during pregnancy” were the most common in case of cholecalciferol, while “fetal exposure during pregnancy”, “maternal exposure during pregnancy”, “premature baby”, “premature delivery”, and “low birth weight baby” were the reported PTs for ergocalciferol. Notably, ergocalciferol showed the highest PRR value for pregnancy, puerperium, and perinatal conditions (19.30; 95%CI 16.12–23.12) followed by calcitriol (9.00; 95%CI 7.14–11.36) (Table 5). Since pharmacovigilance data do not allow for investigation of causality when several drugs are administered concomitantly, all ICSRs containing the abovementioned ADRs were analyzed for each VDR agonist. It was revealed that only 64 ICSRs reported the use of cholecalciferol alone. Of these cases, 10 ICSRs reported severe ADRs.

Further, the potential complications of maternal and fetal exposure during pregnancy can also be found in the congenital, familial, and genetic disorder SOCs, where ergocalciferol exhibit the highest PRR value (11.58; 95%CI 8.40–15.97) followed by calcitriol (3.51; 95%CI 2.12–5.81).

The high values of PRR related to psychiatric disorders also deserved further analysis. For cholecalciferol, the most frequently reported preferred terms (PTs) were confusional state, reported in 360 ICSRs (18.56% of all ICSRs with PTs from the psychiatric disorders SOC), followed by insomnia in 335 ICSRs (17.27%), anxiety in 255 ICSRs (13.14%), depression in 205 ICSRs (10.57%), sleep disorders in 186 ICSRs (9.59%), and adjustment disorders in 98 ICSRs (5.05%). On the other hand, ergocalciferol was linked to sleep disorders in 53 ICSRs (17.04%), to anxiety in 44 ICSRs (14.15%), confusional state (in 38 ICSRs (12.22%), stress in 27 ICSRs (8.68%), depression in 21 ICSRs (6.75%), and insomnia in 19 ICSRs (6.11%).

Analyzing all ICSRs reporting psychiatric conditions, it was found that only 6 cases of insomnia and 3 cases of confusional state could directly be linked to cholecalciferol, and 40 cases of anxiety and depression contained cholecalciferol as a suspected drug. In the remaining cases, a combination of several drugs was reported as a potential cause of ADRs.

## 3. Discussion

This study proposed to analyze the safety profiles of vitamin D receptor agonists using the EudraVigilance database, focusing on the compounds available as food supplements. All ICSRs were extracted and analyzed. To date, this is the first study using the EudraVigilance system to provide real-world safety data on the use of vitamin D agonists.

Several years ago, the EMA opened EudraVigilance to a wider audience to improve safety monitoring, make more data available for research, and facilitate access for healthcare professionals and patients to find out about suspected adverse reactions [25]. Since then, the safety profile of various drug classes (e.g., antiseizure medications, anticoagulants, oncologic biosimilars) has been reassessed and their therapeutic use has been updated [26,27,28,29]. Since the natural compounds, both the precursors (cholecalciferol and ergocalciferol) and the active form (calcitriol), are included in combination drugs and prescribed in various diseases, the EudraVigilance system contains a huge number of ICSRs. Along with natural compounds, synthetic agonists are also listed, which allows the database to be used for a direct comparison of safety profiles between VDR agonists. In this study, more than 80,000 ADRs reported in 17,947 ICSRs were extracted and analyzed for all VDR agonists registered in the European Economic Area. Of these reports, more than two thirds were registered for cholecalciferol followed by calcitriol.

The safety profile of each VDR agonist has been assessed previously based on clinical trial data and meta-analyses [30], but pharmacovigilance data might offer a different insight into their adverse reactions. Recently, Maggini et al. conducted a comprehensive study using the Italian National Pharmacovigilance and Phytovigilance systems on the safety profile of cholecalciferol, which provided new information on the possible risk factors leading to serious adverse reactions [23]. They found that the daily dose of cholecalciferol, the duration of treatment, and drug interactions might contribute to the risk of serious ADRs, whereas age and sex do not have a significant influence. This study confirmed that polypharmacy defined as the concurrent use of five or more drugs along with overdose are the most important contributors to developing serious ADR. However, this is not surprising and applies to all drugs. Nevertheless, the current study showed that males and people between the ages of 18 and 64 are more likely to experience serious ADRs when using VDR agonists. More importantly, the concurrent use of VDR agonists and calcium salts was significantly associated with higher risk of developing serious ADRs.

Hypercalcemia is known as the most feared ADR associated with vitamin D agonists. Indeed, this study confirmed that hypercalcemia is a highly specific complication of VDR receptor activation and all drugs had high disproportionate reporting ratios for metabolism and nutrition disorders, the primary SOC for hypercalcemia. However, the complications of hypercalcemia including renal and urinary disorders, cardiac disorders, and nervous systems disorders were associated with 1α- or 25-hydroxy derivatives (i.e., alfacalcidol, calcitriol, calcifediol) only. Recently published research by Kawai et al. assessed the effects of active vitamin D3 analogs on acute kidney injury using the Japanese Adverse Drug Event Report Database. It was shown that active vitamin D3 is associated with acute kidney injury development, so patients with risk factors should be monitored for renal complications when prescribed these medications [31]. The current study’s findings also support that alfacalcidol and calcitriol can cause severe renal complications, having the highest PRR values for serious ADRs in SOC Renal and urinary disorders. In order to understand the link between VDR agonists and hypercalcemia, the current study implemented a multiple logistic regression analysis, which showed that dehydration and overdose were the most important risk factors for developing hypercalcemia. The concurrent use of lithium, calcium salts, thiazide, and thiazide-like diuretics was also a significant contributor to blood calcium increase. Furthermore, the combination of VDR agonists posed the greatest risk of hypercalcemia. These observations align with previous studies, which confirmed that the presence of several hypercalcemic conditions can result in severe complications. Although the impact of age and sex was negligible, males had a higher risk than females.

Despite the fact that hypercalcemia might cause gastrointestinal symptoms, cholecalciferol and ergocalciferol were more frequently associated with abdominal pain and discomfort than other VDR agonists. The meta-analysis conducted by Malihi et al. did not reveal a significant association between gastrointestinal symptoms and cholecalciferol or ergocalciferol [30], but it should be noted that the studies analyzed by them were conducted on adults (>18 years), whereas the current study analyzed ADRs reported for all age groups. Subgroup analyses of ADR distribution by age have confirmed that gastrointestinal ADRs are more prevalent in infants and children (<18 years) than in adults. This is in accordance with previous results that showed that drug use, in general, is associated with an increased risk of upper gastrointestinal complications in children [32].

Cholecalciferol prescription and use in the general population have seen a huge increase in the last decade, likely linked to the great amount of evidence regarding the potential roles of VDR in various diseases, especially immune system conditions [3,33,34], cardiovascular [35,36], neurological, and psychiatric conditions [37,38,39,40]. Several studies indicate an increase in the number of prescriptions for cholecalciferol and cholecalciferol plus calcium, while for alfacalcidol and calcitriol, a steady or declining trend has been documented [41,42,43]. This study shows that with the growing use of cholecalciferol, the number of ADRs registered in EudraVigilance is steadily increasing. Cases including injury, poisoning, and procedural complications were more likely to occur when cholecalciferol and ergocalciferol were used, as reflected by the PRR values calculated for this SOC. This might also be attributed to the overprescribing and the uncontrolled use and access to food supplements containing high doses of these compounds [44].

The widespread use of cholecalciferol in pregnancy has led to a great number of ADRs reported in SOC pregnancy, puerperium, and perinatal conditions. Although the pharmacovigilance data cannot provide an insight into the incidence of severe ADRs among pregnant patients, it was shown in this study that of all pregnancy related ADRs only a small percentage of 15.2% was linked to cholecalciferol administration alone. The high number of reported PTs such as “maternal exposure during pregnancy” or “fetal exposure during pregnancy” indicated that cholecalciferol administration is commonly used in pregnant women [45] and the disproportionality analysis was affected by a confounding by indication [46]. Specifically designed, prospective studies are needed to further explore the safety of cholecalciferol administration during pregnancy.

The disproportionality analysis provided another possible safety signal for SOC psychiatric disorders. This case was also investigated more in-depth by analyzing ICSRs for possible causality links. It was found that the majority of confusional states and insomnia were reported for patients taking several drugs. Similarly, a high proportion of cases reporting anxiety and depression could not be linked to cholecalciferol administration, and the coincidence of psychiatric conditions during cholecalciferol administration might be related to comorbidities and use as food supplement. Furthermore, recent studies have provided some evidence that cholecalciferol administration might have beneficial effects in psychiatric conditions and neurodegenerative diseases [40,47], which could have had an impact on its prescription in these diseases, and again, this signal might have been influenced by indication.

This study has several limitations. First, because the EudraVigilance database only includes cases in which ADRs occurred and provides no information on the total number of users, the incidence of ADRs could not be calculated. Second, the reliability of data obtained from the EudraVigilance database, similar to other pharmacovigilance systems, might be affected by reporting bias, underreporting, and missing data, which may have affected the PRR and ROR values reported here. However, the robustness and reliability of pharmacovigilance data can be improved by refining reporting practices and data analysis techniques. Third, female patients predominated among those using VDR agonists prescribed to treat or prevent osteoporosis. This predominance might have affected our regression results. Despite the limitations, the authors believe these data are significant and must be interpreted accordingly. Furthermore, the findings of this study may be compared with other pharmacovigilance databases, such as FAERS or VigiBase.

## 4. Materials and Methods

### 4.1. Data Extraction and Management

The EudraVigilance database (www.adrreports.eu) was reviewed retrospectively for public information regarding adverse reactions for VDR agonists (cholecalciferol, ergocalciferol, calcifediol, calcitriol, alfacalcidol, paricalcitol, calcipotriol, dihydrotachysterol, doxercalciferol, and tacalcitol). Individual Case Safety Reports (“ICSRs”) reported in the EudraVigilance database between 1 January 2004 and 23 June 2024 were collected.

To include all available ADR data, we exported the line listings for each substance over the specified study period (http://www.adrreports.eu/en/index.html accessed on 23 June 2024). All individually reported cases (ICSRs) were collected and checked for duplicates. The obtained dataset including all relevant ICSRs was used for analysis. ADRs coded in the preferred term (PT) were transformed into “system organ class” (SOC) through MedDRA Desktop Browser (version 27.1). Since there can be variations in reporting patterns, including the description of ADRs by the reporters, which could result in the use of different PTs for the same ADR, this study treated hypercalcemia and blood calcium increased as equals.

Using PRR as a measure of disproportionality, we compared the safety profile of VDR agonists. To evaluate whether the observed number of reports for an ADR–drug combination is greater than that of other drug–reaction combinations in the database, we performed a frequency analysis using 2 × 2 contingency tables. The ROR, 95% confidence interval (95% CI), *p*-value (statistically significant if *p* < 0.05), and chi-square statistics were calculated [48]. The PRR value was calculated to compare the ADR attributed to the different vitamin D agonists included in the study. The same criteria were used in our research as in the SDR definition used by the EMA in 2006—number of reported cases ≥ 3, chi-square ≥ 4, and PRR ≥ 2. This study used the full dataset comparator for signal detection, with this comparator having the advantage of reducing the impact of reporting biases compared with restricted comparators, but likely introducing confounding variables by indication [46].

### 4.2. Statistical Analysis

The data extraction, curation, and mathematical calculations were performed with Microsoft Excel^®^ (MS Office 365, Microsoft Corp., Redmond, WA, USA) and Python (version 3.10.2; Python Software Foundation, Beaverton, OR, USA). For statistical analysis, the GraphPad Prism 8 software (GraphPad Software Inc.; San Diego, CA, USA) and Python (version 3.10.2) were used. The following Python features were used: pandas library for data management; scipy for the chi-square tests; and statsmodels to calculate post hoc adjusted residuals and corresponding *p*-values for pairwise comparisons. First, we conducted a chi-square test of independence for each outcome category across different compounds. For post hoc analysis, adjusted residuals were computed to identify significant deviations. A Bonferroni correction was applied to account for multiple comparisons across compounds and outcomes. The data were stratified, and a subgroup analysis was performed to evaluate the effect of age, sex, and specific medical conditions. Logistic regression–maximum likelihood estimation (MLE) was performed using the logit function of Python’s statsmodels module. Different MLE models were compared based on their pseudo R-squared, Log-likelihood, and AIC values.

## 5. Conclusions

In conclusion, the pharmacovigilance data presented here support that vitamin D3 and its analogs are strongly associated with increased blood calcium levels and there are several risk factors that contribute significantly to the development of hypercalcemia. The logistic regression analysis demonstrated that the association of VDR agonists, overdosing, concomitant administration of calcium salts, drug interactions with thiazide diuretics and lithium, and dehydration significantly increase hypercalcemia risk. Furthermore, the disproportionality analysis showed that the possible consequences of hypercalcemia such as nephrolithiasis, cardiac disorders, neurological disorders, and pancreatitis were associated with active (i.e., 1α-hydroxylated derivatives) VDR agonists only. These findings underscore the necessity for healthcare providers to meticulously monitor patients receiving 1α-hydroxylated derivatives to prevent adverse outcomes. Regular blood tests to check calcium levels, along with patient education regarding the signs and symptoms of hypercalcemia, are crucial components of patient safety protocols.

## Figures and Tables

**Table 1:** Distribution of individual case safety reports registered for vitamin D receptor agonists between 2004 and 2024 according to age groups.

VDR Agonist/ No. of ICSRs	Not Specified (%)	0–1 Month (%)	2 Months–2 Years (%)	3–11 Years (%)	12–17 Years (%)	18–64 Years (%)	65–85 Years (%)	More than 85 Years (%)	Total
Cholecalciferol	3520 (27.2)	533 (4.12)	406 (3.14)	181 (1.40)	117 (0.90)	3981 (30.8)	3602 (27.8)	604 (4.67)	12,944
Ergocalciferol	177 (21.4)	61 (7.38)	29 (3.51)	11 (1.33)	6 (0.73)	313 (37.9)	209 (25.3)	20 (2.42)	826
Calcifediol	42 (7.45)	2 (0.35)	8 (1.42)	7 (1.24)	0	184 (32.6)	264 (46.8)	57 (1.01)	564
Calcitriol	257 (19.0)	9 (0.66)	23 (1.70)	35 (2.58)	36 (2.66)	494 (36.5)	446 (32.9)	55 (4.06)	1355
Alfacalcidol	131 (11.2)	11 (0.94)	24 (2.06)	35 (3.00)	21 (18.0)	404 (34.7)	460 (39.5)	80 (6.86)	1166
Paricalcitol	131 (20.1)	1 (0.15)	0	1 (0.15)	5 (0.77)	253 (38.9)	244 (37.5)	16 (2.46)	651
Dihydrotachysterol	9 (13.2)	0	0	1 (1.47)	0	33 (48.5)	23 (33.8)	2 (2.94)	68
Doxercalciferol	3 (13.6)	0	0	0	0	13 (59.1)	5 (22.7)	1 (4.55)	22
Tacalcitol	6 (17.7)	0	0	2 (5.88)	0	18 (52.9)	7 (20.6)	1 (2.94)	34
Calcipotriol	84 (26.5)	1 (0.32)	5 (1.58)	3 (0.95)	4 (1.26)	134 (42.3)	75 (23.7)	11 (3.47)	317
Total (%)	4360 (24.3)	618 (3.44)	495 (2.76)	276 (1.54)	189 (1.05)	5827 (32.5)	5335 (29.73)	847 (4.72)	17,947

Legend: ICSR—individual case safety report; VDR—vitamin D receptor.

**Table 2:** Distribution of individual case safety reports registered for vitamin D receptor agonists between 2004 and 2024 according to seriousness criteria.

VDR Agonist/ No. of ICSRs	Results in Death (%)	Life Threatening (%)	Disabling (%)	Caused/Prolonged Hospitalization (%)	Congenital Anomaly (%)	Other Medically Important Condition (%)	Not Specified/Unknown (%)	Total
Cholecalciferol	586 (4.53)	457 (3.53)	207 (1.60)	2015 (15.6)	26 (0.20)	3902 (30.2)	5751 (44.4)	12,944
Ergocalciferol	92 (11.1) *	43 (5.21)	18 (2.18)	279 (33.8) *	1 (0.12)	351 (42.5) *	42 (5.08) *	826
Calcifediol	46 (8.16) *	20 (3.55)	3 (0.53)	168 (29.8) *	1 (0.18)	118 (20.9) *	208 (36.9) *	564
Calcitriol	93 (6.86) *	72 (5.31)	23 (1.70)	481 (35.5) *	6 (0.44)	504 (37.2) *	176 (13.0) *	1355
Alfacalcidol	58 (4.97)	88 (7.55) *	22 (1.89)	500 (42.9) *	4 (0.34)	331 (28.4)	163 (14.0) *	1166
Paricalcitol	181 (27.8) *	33 (5.07)	9 (1.38)	211 (32.4) *	0 (0.00)	114 (17.5) *	103 (15.8) *	651
Dihydrotachysterol	1 (1.47)	11 (16.2)	0 (0.00)	33 (48.5)	0 (0.00)	8 (11.8)	15 (22.1)	68
Doxercalciferol	1 (4.55)	2 (9.09)	0 (0.00)	7 (31.8)	0 (0.00)	11 (50.0)	1 (4.55)	22
Tacalcitol	2 (5.88)	0 (0.00)	3 (8.82)	10 (29.4)	0 (0.00)	3 (8.82)	16 (47.1)	34
Calcipotriol	6 (1.89)	6 (1.89)	12 (3.79)	68 (21.5)	4 (1.26) *	109 (34.4)	112 (35.3)	317
Total (%)	1066 (5.94)	732 (4.08)	297 (1.65)	3772 (21.0)	42 (0.23)	5451 (30.4)	6587 (36.7)	17,947

Legend: ICSR—individual case safety report; VDR—vitamin D receptor; * *p* < 0.05, significantly different compared to cholecalciferol by using chi-square test followed by post hoc analysis.

**Table 3:** Most frequently reported adverse reactions by preferred terms and system organ class.

SOCs and PTs	Cholecalciferol	Calcitriol	Alfacalcidol	Paricalcitol	Calcifediol	Ergocalciferol
Metabolism and nutrition disorders						
Hypercalcemia	722 (1.24%)	304 (5.28%)	322 (8.33%)	40 (2.14%)	85 (6.78%)	49 (0.64%)
Gastrointestinal disorders						
Abdominal pain/discomfort	2162 (3.71%)	84 (1.46%)	39 (1.01%)	18 (0.98%)	28 (2.23%)	88 (1.2%)
Nausea	995 (1.71%)	112 (1.95%)	43 (1.11%)	29 (1.55%)	30 (2.39%)	86 (1.13%)
Diarrhea	927 (1.59%)	48 (0.83%)	23 (0.59%)	24 (1.29%)	20 (1.60%)	54 (0.71%)
Vomiting	662 (1.14%)	63 (1.09%)	40 (1.03%)	21 (1.13%)	17 (1.36%)	55 (0.72%)
Constipation	557 (0.96%)	78 (1.36%)	23 (0.59%)	3 (0.16%)	10 (0.80%)	43 (0.56%)
General disorders and administration site conditions						
Fatigue	746 (1.28%)	35 (0.61%)	38 (0.98%)	13 (0.70%)	15 (1.20%)	120 (1.57%)
Headache	631 (1.08%)	57 (0.99%)	57 (1.47%)	11 (0.59%)	31 (2.47%)	112 (1.47%)
Choking	609 (1.04%)	2 (0.03%)	1 (0.03%)	-	1 (0.08%)	9 (0.12%)
Pain	559 (0.96%)	50 (0.87%)	120 (3.10%)	28 (1.50%)	10 (0.80%)	77 (1.01%)
Dyspnea	514 (0.88%)	47 (0.82%)	33 (0.85%)	36 (1.93%)	8 (0.64%)	108 (1.41%)
Product issues						
Off-label use	758 (1.30%)	69 (1.20%)	15 (0.39%)	1 (0.05%)	7 (0.56%)	62 (0.81%)
Drug ineffective	582 (1.00%)	67 (1.16%)	-	18 (0.97%)	6 (0.48%)	116 (1.52%)
Skin and subcutaneous tissue disorders						
Pruritus	661 (1.13%)	53 (0.92%)	47 (1.22%)	21 (1.13%)	16 (1.28%)	53 (0.69%)
Rash	543 (0.93%)	44 (0.76%)	70 (1.81%)	10 (0.54%)	7 (0.56%)	38 (0.50%)
Urticaria	345 (0.59%)	16 (0.28%)	16 (0.41%)	8 (0.43%)	5 (0.40%)	13 (0.17%)
Erythema	558 (0.96%)	58 (1.01%)	19 (0.49%)	11 (0.59%)	7 (0.56%)	33 (0.43%)
Renal and urinary disorders						
Acute kidney injury	199 (0.34%)	49 (0.85%)	59 (1.53%)	5 (0.27%)	16 (1.28%)	5 (0.07%)
Renal impairment	84 (0.14%)	24 (0.42%)	42 (1.09%)	5 (0.27%)	3 (0.24%)	9 (0.20%)
Renal failure	94 (0.16%)	23 (0.40%)	39 (1.01%)	-	5 (0.40%)	5 (0.07%)
Nephrocalcinosis	60 (0.10%)	42 (0.73%)	27 (0.70%)	-	4 (0.32%)	15 (0.20%)
Nephrolithiasis	144 (0.25%)	26 (0.45%)	11 (0.28%)	-	4 (0.32%)	8 (0.10%)
Pregnancy puerperium and perinatal conditions						
Maternal exposure during pregnancy	153 (0.26%)	20 (0.35%)	11 (0.28%)	-	1 (0.08%)	18 (0.24%)
Fetal exposure during pregnancy	94 (0.16%)	35 (0.61%)	7 (0.18%)	-	4 (0.32%)	59 (0.77%)
Psychiatric disorders						
Confusional state	360 (0.62%)	24 (0.42%)	35 (0.91%)	11 (0.59%)	7 (0.56%)	38 (0.50%)
Insomnia and sleeping disorder	521 (0.89%)	25 (0.43%)	14 (0.36%)	2 (0.11%)	4 (0.32%)	72 (0.94%)
Anxiety	255 (0.44%)	12 (0.21%)	3 (0.08%)	4 (0.21%)	4 (0.32%)	44 (0.58%)

**Table 4:** The estimated coefficient of independent variables using the best-fit logistic regression model.

Variables	Coefficient	Standard Error	z	*p* > |z|
Intercept	−2.6774	0.098	−27.207	0.000
Age group	0.1810	0.103	1.755	0.079
Sex	−0.2026	0.063	−3.234	0.001
Overdose	1.2878	0.124	10.353	0.000
Calcium supplements	0.7763	0.111	7.021	0.000
Cancer	−0.8620	0.254	−3.399	0.001
Thiazide and thiazide-like diuretics	0.3753	0.110	3.400	0.001
Lithium salts	0.9759	0.372	2.621	0.009
Dehydration	1.6194	0.369	4.389	0.000
Polypharmacy	−0.7028	0.119	−5.919	0.000
Interaction of age–calcium salt	−0.6229	0.131	−4.740	0.000
Interaction of age–dehydration	0.6346	0.426	1.490	0.136
Interaction of age–polypharmacy	0.8343	0.138	6.048	0.000

**Table 5:** Proportional reporting ratio values of specific VDR agonists across all SOCs.

	Cholecalciferol	Calcitriol	Alfacacidol	Paricalcitol	Ergocalciferol	Calcifedol	Cholecalciferol	Calcitriol	Alfacalcidol	Paricalcitol	Ergocalciferol	Calcifedol
System of Class/Vitamin D Agonist	All ADRs						Serious ADRs					
Blood and lymphatic system disorders	0.53	1.04	1.04	0.45	1.12	0.32	0.81	1.11	1.12	0.47	1.09	0.46
Cardiac disorders	1.18	1.12	0.82	2.59	2.70	0.68	1.35	0.94	0.69	2.38	2.18	0.67
Congenital, familial, and genetic disorders	2.92	3.51	1.86	0.00	11.58	1.44	3.56	2.78	1.50	0.00	8.38	1.51
Ear and labyrinth disorders	1.48	0.30	1.42	0.81	1.48	0.88	2.33	0.38	1.51	0.82	1.70	0.44
Endocrine disorders	1.10	2.57	2.21	0.55	5.35	0.36	1.33	2.13	1.86	0.47	3.77	0.40
Eye disorders	1.40	1.29	0.55	0.19	5.37	0.54	1.62	1.17	0.41	0.20	4.73	0.25
Gastrointestinal disorders	2.30	1.18	1.20	0.92	2.78	0.94	2.06	1.14	1.12	0.92	2.89	0.63
General disorders and administration site conditions	0.75	0.68	0.57	0.64	1.53	0.49	1.11	0.81	0.70	0.81	1.87	0.58
Hepatobiliary disorders	2.95	2.26	3.70	0.77	4.62	0.15	3.52	1.77	2.90	0.64	3.37	0.16
Immune system disorders	2.72	1.88	1.56	1.00	8.26	0.49	3.09	1.53	1.20	0.78	6.48	0.14
Infections and infestations	0.75	0.74	0.64	1.11	2.65	0.47	0.98	0.66	0.58	0.99	2.17	0.47
Injury, poisoning, and procedural complications	2.69	1.65	1.44	1.15	3.86	1.84	2.85	1.37	1.16	0.83	3.13	1.55
Investigations	1.42	1.71	1.51	1.44	3.48	0.70	1.72	1.52	1.34	1.05	3.04	0.57
Metabolism and nutrition disorders	2.89	7.75	7.35	3.07	4.30	4.73	3.54	6.65	6.37	2.76	3.47	4.73
Musculoskeletal and connective tissue disorders	1.03	0.66	0.67	0.48	1.99	0.50	1.86	0.92	0.86	0.64	2.77	0.43
Neoplasms: benign, malignant, and unspecified	0.62	0.45	0.45	0.53	2.16	0.15	0.73	0.35	0.35	0.42	1.51	0.15
Nervous system disorders	0.84	0.66	0.70	0.67	1.69	0.65	1.19	0.79	0.79	0.85	2.00	0.61
Pregnancy, puerperium, and perinatal conditions	4.51	9.00	3.75	0.55	19.30	1.90	5.47	6.83	2.99	0.47	13.99	1.76
Product issues	5.95	3.61	1.49	0.51	6.64	1.34	6.58	4.53	1.07	0.00	8.59	0.95
Psychiatric disorders	2.26	1.41	1.10	0.81	5.07	0.46	2.61	1.28	0.99	0.76	4.59	0.42
Renal and urinary disorders	1.96	4.32	4.58	1.30	2.70	2.53	2.01	3.33	3.71	1.08	2.00	2.25
Reproductive system and breast disorders	0.33	0.68	0.19	0.27	1.81	0.23	0.97	1.53	0.48	0.60	4.06	0.25
Respiratory, thoracic, and mediastinal disorders	1.72	0.80	1.02	1.12	3.89	0.49	2.14	0.72	0.91	1.05	3.39	0.42
Skin and subcutaneous tissue disorders	1.81	1.18	1.31	1.04	2.44	0.65	2.09	1.09	1.32	1.03	2.70	0.42
Social circumstances	2.16	0.50	0.85	0.35	8.67	0.17	2.73	0.29	0.67	0.31	6.88	0.20
Surgical and medical procedures	0.66	0.56	0.41	1.52	1.24	2.75	0.82	0.49	0.37	1.32	0.98	3.05
Vascular disorders	1.26	0.96	0.81	1.98	4.32	0.47	1.55	0.84	0.68	1.77	3.62	0.43

Legend: the proportional reporting ratio values greater than 6 (red), 4 (orange), and 2 (pink) were highlighted.

## Data Availability

All data used in the study was obtained using the EudraVigilance database (www.adrreports.eu). The datasets generated and analyzed are available at Mendeley Data http://doi.org/10.17632/287j6mrtyp.1 (accessed on 23 June 2024).

## Supplementary Materials

The following supporting information can be downloaded at: https://www.mdpi.com/article/10.3390/ph17121686/s1, Table S1: Distribution of individual case safety reports submitted to the EudraVigilance database from 2004 to 2024, categorized by reporter type; Table S2: Distribution of individual case safety reports submitted to the EudraVigilance database from 2004 to 2024, categorized by primary source country; Table S3: Distribution of individual case safety reports submitted to the EudraVigilance database from 2004 to 2024, categorized by sex; Table S4: The list of covariates used for logistic regression analysis and the Akaike Information Criterion (AIC) values for all tested models.
