# Supplementary material for: Comparative Analyses of the Safety Profiles of Vitamin D Receptor Agonists: A Pharmacovigilance Study Based on the EudraVigilance Database
Source: Pharmaceuticals (Basel). 2024 Dec 13;17(12):1686. doi: 10.3390/ph17121686 (PMC11677518; doi:10.3390/ph17121686)
Supplement: Supplementary file 1 [file pharmaceuticals-17-01686-s001.zip › pharmaceuticals-3366889-supplementary.pdf]

Supplementary Materials

Table S1. Distribution of individual case safety reports submitted to the EudraVigilance database from 2004 to 2024, categorized by reporter type

No. of reported cases	HCP	%	non-HCP	%	Not specified /unknown	%	Total
Cholecalciferol	7,531	58.18	5,378	41.55	35	0.27	12,944
Ergocalciferol	690	83.54	134	16.22	2	0.24	826
Calcifedol	460	81.56	102	18.09	2	0.35	564
Calcitriol	1,118	82.51	220	16.24	17	1.25	1,355
Alfacalcidol	1,042	89.37	104	8.92	20	1.72	1,166
Paricalcitol	579	88.94	44	6.76	28	4.30	651
Dihydrotachysterol	64	94.12	2	2.94	2	2.94	68
Doxercalciferol	22	100.00	0	0.00	0	0.00	22
Tacalcitol	26	76.47	5	14.71	3	8.82	34
Calcipotriol	239	75.39	69	21.77	9	2.84	317
Total	**11,771**	**65.59**	**6,058**	**33.75**	**118**	**0.66**	**17,947**

Legend: HCP – healthcare professionals.

Table S2. Distribution of individual case safety reports submitted to the EudraVigilance database from 2004 to 2024, categorized by primary source country

No. of reported cases	EEA	%	non-EEA	%	Total
Cholecalciferol	8,369	64.66	4,575	35.34	12,944
Ergocalciferol	173	20.94	653	79.06	826
Calcifedol	424	75.18	140	24.82	564
Calcitriol	432	31.88	923	68.12	1,355
Alfacalcidol	569	48.80	597	51.20	1,166
Paricalcitol	423	64.98	228	35.02	651
Dihydrotachysterol	58	85.29	10	14.71	68
Doxercalciferol	0	0.00	22	100.00	22
Tacalcitol	19	55.88	15	44.12	34
Calcipotriol	193	60.88	124	39.12	317
Total	**10,660**	**59.40**	**7,287**	**40.60**	**17,947**

Legend: EEA – European Economic Area.

Table S3. Distribution of individual case safety reports submitted to the EudraVigilance database from 2004 to 2024, categorized by gender

No. of reported cases	Male	%	Female	%	Not specified/ unknown	%	Total
Cholecalciferol	3,021	23.34	9,448	72.99	475	3.67	12,944
Ergocalciferol	181	21.91	606	73.37	39	4.72	826
Calcifedol	165	29.26	388	68.79	11	1.95	564
Calcitriol	435	32.10	856	63.17	64	4.72	1,355
Alfacalcidol	356	30.53	778	66.72	32	2.74	1,166
Paricalcitol	309	47.47	308	47.31	34	5.22	651
Dihydrotachysterol	9	13.24	57	83.82	2	2.94	68
Doxercalciferol	14	63.64	8	36.36	0	0.00	22
Tacalcitol	19	55.88	14	41.18	1	2.94	34
Calcipotriol	163	51.42	146	46.06	8	2.52	317
Total	**4,672**	**26.03**	**12,609**	**70.26**	**666**	**3.71**	**17,947**

Table S4. The list of covariates used for logistic regression analysis and the Akaike Information Criterion (AIC) values for all tested models

Models	Covariates	Number of covariates	AIC
Model 1	Overdose (absent = 0, present = 1); Calcium supplements (absent = 0, present = 1); Cancer (absent = 0, present = 1); Thiazide and thiazide-like diuretics (absent = 0, present = 1); Lithium salts (absent = 0, present = 1); Dehydration (absent = 0, present = 1); Polypharmacy (less than 5 = 0, more or equal than 5 = 1)	6	8493.8
Model 2	Overdose (absent = 0, present = 1); Calcium supplements (absent = 0, present = 1); Cancer (absent = 0, present = 1); Thiazide and thiazide-like diuretics (absent = 0, present = 1); Lithium salts (absent = 0, present = 1); Dehydration (absent = 0, present = 1); Polypharmacy (less than 5 = 0, more or equal than 5 = 1)	7	9102.2
Model 3	Age group (18-64 years = 0, <18 and >65 = 1); Sex (male = 0, female = 1); Overdose (absent = 0, present = 1); Calcium supplements	9	8471.8

	(absent = 0, present = 1); Cancer (absent = 0, present = 1); Thiazide and thiazide-like diuretics (absent = 0, present = 1); Lithium salts (absent = 0, present = 1); Dehydration (absent = 0, present = 1); Polypharmacy (less than 5 = 0, more or equal than 5 = 1)		
Model 4	Age group (18-64 years = 0, <18 and >65 = 1); Sex (male = 0, female = 1); Overdose (absent = 0, present = 1); Calcium supplements (absent = 0, present = 1); Cancer (absent = 0, present = 1); Thiazide and thiazide-like diuretics (absent = 0, present = 1); Lithium salts (absent = 0, present = 1); Dehydration (absent = 0, present = 1); Polypharmacy (less than 5 = 0, more or equal than 5 = 1); Interaction age:calcium salt; Interaction age:dehydration; Interaction age:polypharmacy	12	8391.8
Model 5	Age group (18-64 years = 0, <18 and >65 = 1); Sex (male = 0, female = 1); Overdose (absent = 0, present = 1); Calcium supplements (absent = 0, present = 1); Cancer (absent = 0, present = 1); Thiazide and thiazide-like diuretics (absent = 0, present = 1); Lithium salts (absent = 0, present = 1); Dehydration (absent = 0, present = 1); Polypharmacy (less than 5 = 0, more or equal than 5 = 1); Interaction age:calcium salt; Interaction age:dehydration; Interaction age:polypharmacy; Interaction thiazide and thiazide-like diuretics:dehydration	13	8426.4
Model 6	Age group (18-64 years = 0, <18 and >65 = 1); Sex (male = 0, female = 1); Overdose (absent = 0, present = 1); Calcium supplements (absent = 0, present = 1); Cancer (absent = 0, present = 1); Thiazide and thiazide-like diuretics (absent = 0, present = 1); Lithium salts (absent = 0, present = 1); Dehydration (absent = 0, present = 1); Polypharmacy (less than 5 = 0, more or equal than 5 = 1); Interaction age:overdose; Interaction age:calcium salt; Interaction age:dehydration; Interaction age:polypharmacy; Interaction overdose:calcium salts; Interaction overdose:thiazide and thiazide-like diuretics; interaction overdose:lithium salts; interaction calcium salts:thiazide and thiazide-like diuretics; interaction calcium salts:lithium salts; interaction thiazide and thiazide-like diuretics:lithium salts.	19	8429.2
